# Interactions of pathogenic *Escherichia coli* with CEACAMs

**DOI:** 10.3389/fimmu.2023.1120331

**Published:** 2023-02-14

**Authors:** Alaullah Sheikh, James M. Fleckenstein

**Affiliations:** ^1^ Division of Infectious Diseases, Department of Medicine, Washington University School of Medicine, St. Louis, MO, United States; ^2^ Infectious Diseases, Medicine Service, Veterans Affairs Saint Louis Health Care System, Saint Louis, MO, United States

**Keywords:** Escherichia coli, ETEC (enterotoxigenic Escherichia coli), CEACAM, Afa/dr family adhesins, type 1 fimbriae

## Abstract

The pathogenic *Escherichia coli* can be parsed into specific variants (pathovars) depending on their phenotypic behavior and/or expression of specific virulence factors. These pathogens are built around chromosomally-encoded core attributes and through acquisition of specific virulence genes that direct their interaction with the host. Engagement of *E. coli* pathovars with CEACAMs is determined both by core elements common to all *E. coli* as well as extrachromosomally-encoded pathovar-specific virulence traits, which target amino terminal immunoglobulin variable-like (IgV) regions of CEACAMs. Emerging data suggests that engagement of CEACAMs does not unilaterally benefit the pathogen and that these interactions may also provide an avenue for pathogen elimination.

## Introduction

Pathogenic *Escherichia coli* exhibit extraordinary adaptation to multiple host niches, due in part to their remarkable genetic plasticity that has permitted the acquisition of a diverse array of virulence molecules. These pathogens are incredibly diverse as illustrated by whole genome sequencing studies which highlight the open nature of the *E. coli* pangenome (1). In essence, no two isolates of *E. coli* from different sources are likely to be “identical”. Despite this diversity, an important element of niche adaptation among pathogenic *E. coli* is the interaction of their respective adhesins with the family of eukaryotic molecules known as the Carcinoembryonic Antigen -related Cell Adhesion Molecules or CEACAMs. These molecules, encoded on human chromosome 19q13 (2), are cell surface proteins that participate in homotypic intercellular adhesion interactions. CEACAMs are present on many cell types including those lining intestinal and urogenital mucosae where *E. coli* pathogenic variants (pathovars) establish a niche, as well as immune cells which defend these tissues.

CEACAMs share a common architecture in which the amino terminal region of the protein forms a domain resembling the immunoglobulin variable region (IgV-like), while the remainder of extracellular portion of each protein is formed by a variable number of domains similar to immunoglobulin constant regions (3). Homotypic dimerization is primarily directed by the interaction of the N-terminal domains (4). The proteins are also heavily glycosylated along their lengths. Among the intestinal CEACAMs, the C terminal end of CEACAM1 is comprised of a transmembrane region and a cytoplasmic tail in which individual isoforms may contain Immunoreceptor Tyrosine-based Inhibitory Motifs (ITIM), while CEACAMs 5,6, and 7 each end in glycosylphosphotidyl inositol (GPI) anchors.

## Biology of *E. coli* adhesin/CEACAM interactions

**Figure 1:**
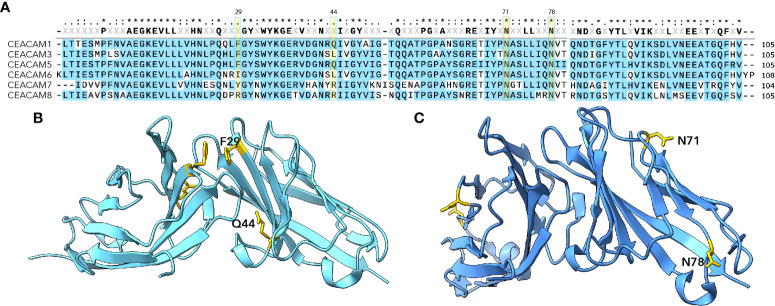
N-terminal IgV-like domains of CEACAMs **(A)** MUSCLE alignment of N-terminal CEACAM domains. Highlighted residues Phe 29 and Gln44 indicate amino acids shown to be critical for Afa/Dr interaction (8); residues at Asn71 and Asn78 indicate glycosylation sites important for interaction with type 1 pili (9) and the FimH adhesin. **(B)** Dimeric CEACAM5 N-terminal domain (PDB 2QSQ) (8) depicting location of Afa/Dr adhesin interactions. **(C)** Dimeric CEACAM6 N-terminal domain (PDB 4Y8A) (4) depicting location of sites critical for interaction with type 1 pili (9).

CEACAMs are among the most rapidly evolving proteins in humans. As the extracellular regions of these proteins serve as receptors for a number of important human pathogens, they appear to be under considerable adaptive pressure (5–7) driving CEACAM polymorphisms as well as species-specific diversity (5). Pathogenic *E. coli* are among the pathogens known to target CEACAM domains exposed on mucosal surfaces. Interestingly, the amino terminal IgV like domains of CEACAMs (**Figure 1A**) are defined targets for several adhesins expressed by the *E. coli* pathovars. To date, two major groups of structures- the Afa/Dr adhesins and type 1 pili have been shown to engage the CEACAM N-terminal domains (**Table 1**).

**Table 1:** Interaction of pathogenic *E. coli* and their ligands with specific CEACAMS.

pathovar	ligand	receptor(s)	type	reference(s)
DAEC	Afa/Dr family adhesins	CEACAMs 1,5,6	protein-protein	(8, 10, 11)
AIEC	type 1 fimbriae/FimH	CEACAM6	lectin	(12–14)
ETEC	type 1 fimbriae/FimH	CEACAM6	lectin	(9, 15, 16)
UPEC	Afa/Dr adhesins	CEACAM5	protein-protein	(17)

The Afa/Dr family of adhesins mediate mannose-resistant hemagglutination and adhesion. Members of this group of adhesion molecules share an operon organization that includes genes encoding a chaperone, an outer membrane protein (usher), and the adhesin. Afa operons encode an AfaB chaperone and an AfaC outer membrane protein, while AfaE molecules serve as afimbrial adhesins. Similarly, Dr systems are comprised of a DraB chaperone, a DraC usher, and a DraE protein which assemble to form thin homopolymeric fimbriae (18–20). Both AfaE as well as DraE can mediate interactions with CEACAMs as receptors (21).

Type 1 pili are ~1 µm long projections from the surface of *E. coli* that are also assembled through a chaperone usher pilus (CUP) pathway which directs the biogenesis of pili comprised of ~1000 copies of the major pilin subunit (FimA), and single copies of FimF and FimG adapter proteins that present the terminal FimH mannose-binding tip adhesin (22). All *E. coli* express chromosomally-encoded type 1 pili which mediate adhesion to mannosylated glycoproteins including CEACAMs. Early studies by Leusch et al, indicated that *E. coli* engage mannosylated residues on CEACAM5 and CEACAM6 (23–25).

Type 1 pili, long known to direct mannose-sensitive adhesion, bind to specific glycosylated residues which are projected on the exposed surface of the CEACAM6 (previously referred to as nonspecific cross-reacting antigen or NCA) (**Figure 1**) (9, 15). In contrast, the Afa/Dr adhesins are thought to engage residues on the inner dimerization face of CEACAMs (10) and are capable of disrupting CEACAM multimers (8).

## Importance of CEACAM-*E. coli* interactions

### Adherent-invasive *E. coli* and the pathogenesis of inflammatory bowel disease

Although the pathogenesis of inflammatory bowel disease is still being dissected, a prevailing concept is that aberrant interactions between intestinal microbes and the immune system are operative. Among the potential contributors to the pathogenesis of Crohn’s disease are the interactions between the host and *E. coli* known as adherent/invasive *E. coli* (AIEC). Although AIEC appear to lack canonical virulence factors found in other gastrointestinal *E. coli* pathovars (26), whole genome sequencing of isolates from patients with Crohn’s disease suggest that they are phylogenetically distinct from commensal *E. coli* (27). AIEC have the ability to engage CEACAM6 *via* FimH (28), with some AIEC having acquired pathoadaptive mutations within the *fimH* gene that augment interactions between FimH and CEACAM6, and which enhance the propensity of AIEC to induce intestinal inflammation (12). Enhanced expression of CEACAM6 in individuals with inflammatory bowel disease promoted by inflammatory mediators accelerates binding of AIEC (13, 29, 30), and AIEC infection of epithelial cells *in vitro* induces expression of multiple CEACAMs (31). Dumych, et al. also proposed that CEACAMs expressed on early apoptotic cells exhibit high mannose glycosylation at specific sites (Asn197, and Asn 224) and that FimH can induce the formation of membrane blebs that present these immature high-mannose glycans on their surface (32). Conversely, mannoside compounds which antagonize FimH -CEACAM6 interactions may interrupt AIEC colonization and mitigate subsequent inflammatory changes (14).

Altogether, however the precise role played by AIEC in the pathogenesis of Crohn’s disease is uncertain (33). Indeed, the pathogenesis of inflammatory bowel disease is complex, potentially involving not only AIEC, but human susceptibility genes including a loss of function variant of the protein tyrosine phosphatase non-receptor type 2 (*PTPN2*) gene (34). Notably, PTPN2 appears to modulate the gut microbiome (35) and loss of PTPN2 is associated with enhanced CEACAM expression and enhanced AIEC uptake and intracellular survival (36), suggesting that Crohn’s disease may reflect the interplay of pathogenic *E. coli* and distinctly susceptible hosts.

### Diffusely adherent *E. coli*

The diffusely adhering *E. coli* (DAEC) are a diverse pathovar that have been associated with gastrointestinal (37–39) as well as urinary tract (40, 41) infections (42, 43). From the small number of whole genome sequences of these strains presently available, DAEC appear to be phylogenetically close to enteroaggregative *E. coli*, but are distinguished in their complement of adhesins as well as iron acquisition systems (44). Although DAEC have been isolated from patients with ulcerative colitis (UC) (45), their role in the molecular pathogenesis of UC remains unclear (33). These strains are defined phenotypically by their diffuse adherence pattern on cultured epithelial cells, and genetically by the expression of a group of virulence molecules known collectively as Afa/Dr adhesins which may constitute either fimbrial or afimbrial (Afa) structures which bind to the human decay accelerating factor (hDAF, also known as CD55) a glycosylphosphatidylinositol (GPI)-anchored glycoprotein present on many cell types including epithelial cells. A subset of this family of adhesins engage CEACAMs including CEACAM1, CEACAM5 (10, 46), and CEACAM6, which are recruited to the sites of bacterial attachment (46), along with lipid raft markers (47). CEACAM1-4L, a splice variant of CEACAM1, has an extended cytoplasmic L domain that can modulate cellular signaling following DAEC engagement (44). Engagement of either of the GPI-anchored CEACAM5 or CEACAM6 molecules can drive efficient internalization of Dr-fimbriated DAEC (21, 47). Interestingly, increased expression of Afa/Dr fimbriae has recently been associated with the emergence of the multidrug-resistant ST131 uropathogenic (UPEC) clones (48). Although UPEC expressing Afa/Dr fimbriae represent less than 10% of UTI isolates, engagement of CEACAMs in the urogenital epithelia suppresses exfoliation of the epithelial cells and enhances colonization (17). Notably, more recent studies demonstrate that CEACAM engagement by pathogenic bacteria results in delivery of bacterial nitric oxide that activates eukaryotic cGMP-dependent signaling pathways to enhance expression of CD105 (endoglin) (49). Increases in CD105 expression in turn abrogate detachment of cells targeted by bacteria by increasing their affinity for extracellular matrix (50).Therefore, engagement of CEACAMs could benefit the pathogen by facilitating colonization while suppressing host mechanisms for elimination.

### Enterotoxigenic E. coli (ETEC)

The enterotoxigenic *E. coli* (ETEC) are a diverse pathovar defined by the production of heat-labile toxin (LT) and/or heat-stable toxins (ST). These pathogens are a predominant cause of acute diarrheal illness as well as deaths due to diarrhea in developing countries among young children. Likewise, ETEC are perennially the major cause of traveler’s diarrhea (51, 52). ETEC have also been linked repeatedly to poorly understood sequelae among young children in LMICs including enteropathic changes to the small intestine and accompanying nutrient malabsorption and growth stunting (53–57).

The basic mechanisms by which ETEC enterotoxins cause diarrhea are known (58). Both toxins activate major cyclic nucleotide second messenger pathways in the cell. LT, like cholera toxin, stimulates adenylate cyclase resulting in increases in intracellular cAMP which in turn activates protein kinase A (PKA). PKA-mediated phosphorylation modulates the activity of cellular ion channels including the cystic fibrosis transmembrane regulator (CFTR) and the sodium-hydrogen ion exchanger (NHE3), resulting in the net export of salt and water into the intestinal lumen and watery diarrhea typical of ETEC. ST, similar to endogenous gastrointestinal peptides guanylin and uroguanylin, binds to guanylate cyclase C resulting in the increased production of cGMP. Increases in cGMP in turn activate Protein kinase G (PKG) which likewise phosphorylates and modulates ion channels resulting in diarrhea.

In contrast, the molecular basis of sequelae associated with ETEC, and the contribution of enterotoxins to enteropathic changes linked to ETEC remains enigmatic. Notably, cAMP and PKA are known to modulate hundreds of eukaryotic genes (59). Binding of cAMP to PKA, a heterotetramer comprised of two regulatory and two catalytic subunits, in the cytoplasm liberates PKA catalytic subunits to enter the nucleus (60) where they phosphorylate the cyclic AMP response element binding protein (CREB) at position S-133 (61, 62). The activated CREB transcription factor is then free to engage multiple cAMP-responsive elements (CRE, *e.g*., 5’-TGACGTCA-3’) in the regulatory regions of approximately 4000 genes within the human genome. Not surprisingly, recent studies of transcriptional modulation by LT have revealed that it impacts the expression of hundreds of genes in intestinal epithelial cells (63).

Among the many genes modulated by LT are those encoding CEACAMs expressed within the gastrointestinal tract. Although CEACAM expression is normally more robust in the colon, the expression of CEACAMs5, 6, and 7 in small intestinal epithelia are all substantially upregulated by LT as well as forskolin which also stimulates production of cAMP (16). Of note however, promoter regions of these genes appear to lack canonical CRE sites suggesting cAMP-mediated upregulation of their expression is indirect. This increased expression of CEACAM6 enhanced ETEC adhesion to target intestinal epithelial cells, with ETEC recruited specifically to regions of increased CEACAM expression. Conversely deletion of CEACAM6 by CRISPR-Cas9 resulted in a marked decrease in ETEC adhesion while restoration of CEACAM6 expression rescued the adhesion phenotype. Similarly, heterologous expression of CEACAM6 in HeLa cells resulted in marked increases in ETEC adhesion. Moreover, small intestinal biopsies of ETEC infected patients also demonstrated significant increases in CEACAM6 expression following infection. Together, these studies suggested that CEACAMs serve as important receptors for ETEC and that these pathogens exploit cAMP-dependent cellular pathways to alter CEACAM expression in establishing their niche in the small intestine.

Moreover, we demonstrated that FimH, the tip adhesin of type 1 pili, interacts specifically with CEACAM6 in a mannose-dependent fashion. Collectively, these data suggest that ETEC, *via* its plasmid-encoded heat-labile toxin stimulates the production CEACAMs to augment pathogen-host interactions mediated by chromosomally-encoded type 1 pili. In effect, ETEC alter the epithelial landscape, at least transiently, to suit the bacteria. Many important questions remain however, including whether these changes in CEACAM expression also impact other organisms including commensal *E. coli* and other *E. coli* pathovars that could also engage CEACAMs.

### CEACAMs as innate defense molecules

The role of CEACAMs that are shed from intestinal epithelia and which could act as molecular decoys for pathogenic microbes remains largely unexplored. Nevertheless, these molecules have the potential to modulate pathogen-host interactions, particularly in the gastrointestinal tract. Large amounts of CEACAM5, estimated to be 50-70 mg, is normally shed in human feces (64–67) with the majority appearing in membrane-bound forms that can be released by cleavage of the GPI anchor with phosphatidylinositol-specific phospholipase C (PI_PLC). Studies of primary colonic epithelial cells in culture indicated that soluble CEACAMs may also be released by endogenous phospholipases (68). Although the precise molecular mechanisms by which CEACAMs are shed into the intestinal lumen and other mucosal spaces have not been definitely established, early studies of transformed intestinal epithelial cells indicated that CEACAMs are released in a directed fashion, specifically from the apical brush border surface (69). Subsequently it has been noted that HT-29 cells release significant amounts of extracellular vesicles (ECV) bearing CEACAMs on their surface, particularly when the cells are under stress (70). It has also been suggested that microvilli, which form the intestinal brush border, release ECV into the lumen (71, 72). Intriguingly, ECV (73) actively released from the tips of microvilli (74) comprising the intestinal brush border contain enzymatically active proteins, and can mitigate interactions of pathogenic *E. coli* with target epithelia (75). It has also been suggested that intestinal M cells express both CEACAM5 and CEACAM1 on their apical glycocalyx, but these cells differ from enterocytes in lacking the formation of CEACAM-laden vesicles (76). Others have indicated that CEACAM5 and CEACAM6 may be produced by goblet cells. (72, 77) and this appears to be supported by available human small intestine single cell RNAseq data (78).

It has also been suggested that innate responses to pathogen interaction can lead to induction of cytokines that alter expression of CEACAMs by intestinal epithelia, such as the induction of CEACAM5 and CEACAM6 by INF-γ (79). Conversely, others have argued that Gram-negative organisms might be able to impair release of CEA from intestinal epithelia (80).

## Conclusion

The expression of a variety of CEACAMs by multiple cell types including those lining mucosal surfaces such as the gastrointestinal tract or on immune effector cells suggests that pathogenic *E. coli* are likely to encounter these molecules in their transit through human hosts. At present however, despite the substantial diversity of pathogenic *E. coli* our understanding of the contribution of these molecules in *E. coli* pathogen-host interactions is limited to a few select pathovars. Already, however, some important themes have emerged from studies of the molecular interactions between pathogen and host. *E. coli* appear to have adopted several strategies to engage CEACAMs. Ongoing studies are likely to further illuminate the critical nature of these interactions in directing the outcome of several important human infections.

## Author contributions

Both authors contributed to the literature review. JF drafted the manuscript and figures. All authors contributed to the article and approved the submitted version.
